# Evaluation of Viral Collection Efficiency with Antibody-Modified Magnetic Particles by Polymerase Chain Reaction Assay

**DOI:** 10.3390/s26031019

**Published:** 2026-02-04

**Authors:** Masato Yasuura, Hiroki Ashiba, Ken-ichi Nomura

**Affiliations:** Sensing Technology Research Institute, National Institute of Advanced Industrial Science and Technology (AIST), Central 5, 1-1-1 Higashi, Tsukuba 305-8565, Ibaraki, Japan; h.ashiba@aist.go.jp (H.A.); k-nomura@aist.go.jp (K.-i.N.)

**Keywords:** influenza virus, immunoassay, bead-based assay, highly sensitive detection, digital assay, rapid detection

## Abstract

Polymerase chain reaction (PCR) is the primary method for virus detection; however, its complex preprocessing has prompted research into simpler immunoassay-based approaches. Among these, techniques using antibody-modified magnetic particles, exemplified by digital ELISA, provide ultra-high sensitivity comparable to PCR by efficiently capturing trace viruses and enabling concentration, washing, and transfer to microreactors. In this study, we evaluated the virus capture efficiency of antibody-modified magnetic particles based on quantitative PCR (qPCR). Influenza A virus (H1N1/A/Puerto Rico/8/1934) was tested with 1 μm magnetic beads modified with HA1 antibodies. As quantification becomes unreliable and difficult in an extremely low-concentration range near the detection limit of qPCR, low-concentration viral suspensions (10^5^ copies/mL) were mixed with particle dispersions (up to 5 × 10^8^ particles/mL) for 10 min, followed by magnetic separation and washing, and the remaining virus in each fraction was analyzed by qPCR. At the highest particle concentration, capture rates exceeded 80% relative to the initial suspension, indicating near-complete capturing when considering free nucleic acids. Time-course analysis showed that the capture rate reached saturation within 2 min, with approximately 90% of the saturation at 1 min. Furthermore, kinetic modeling of magnetic bead–virus binding reproduced experimental data. These findings demonstrate that short mixing times with high particle concentrations enable efficient virus capture, contributing to the development of rapid and highly sensitive immunoassay systems.

## 1. Introduction

The COVID-19 pandemic highlighted the need for further advancements in rapid and reliable virus detection methods. Although numerous SARS-CoV-2 diagnostic kits [1,2,3,4,5,6,7,8,9,10,11] have been authorized for clinical use and self-testing, simple antigen assays based on immunochromatography [2,3,4,12,13] remain widely adopted due to their speed (approximately 10–20 min). However, their limits of detection (LoD) are substantially higher than those of quantitative antigen assays [5,6,7] and polymerase chain reaction (PCR) tests [9,10,11], often necessitating confirmatory PCR testing for negative results [14,15]. PCR is the gold standard based on its sensitivity and specificity [14,15], yet its operational complexity and long turnaround times restrict its utility for point-of-care (POC) screening during pandemics. Consequently, there is a critical need for antigen tests that combine simplicity and rapidity with PCR-level sensitivity.

Recent advances in immunoassay-based detection [2,3,4,5,6,7,16,17,18,19,20,21,22], including chemiluminescent enzyme immunoassay (CLEIA) [5,6,7,16,17,18] and digital ELISA (d-ELISA) [19,20,21], have demonstrated promising improvements in sensitivity. Many of these approaches employ antibody-modified magnetic particles (AM-MPs) as multifunctional tools for target capture, concentration, washing, and transfer [17,18,19,20,21,22,23,24,25,26,27]. These developments represent one category of the broader efforts—including immunoassays—to enhance the sensitivity–speed trade-off in detecting viral particles in biological samples by capturing and concentrating them using microbeads (0.5–500 μm), as outlined in our previous report [28]. While non-magnetic microbeads combined with optical readout currently constitute the most widely used strategy, recent studies have increasingly explored magnetic particles, integrating them with electrochemical detection [29] or optical detection to achieve enhanced performance. In particular, magnetic-particle-based methods in immunoassays, such as the aforementioned d-ELISA, have advanced the detection sensitivity to the level nearly comparable to qPCR; however, further development is still required for their practical application as rapid, point-of-care diagnostic tools. Efficient viral capture and concentration are pivotal for achieving rapid and sensitive detection, yet practical implementation requires minimizing reaction times without compromising performance. To address this challenge, we systematically investigated the relationship between AM-MP concentration, stirring time, and viral collection efficiency using quantitative PCR (qPCR). Influenza A virus (IAV), a pathogen historically responsible for pandemics [30], was selected as the model target due to the availability of commercialized antibodies [20,25]. To examine the system from both experimental and kinetic model perspectives, it would be ideal to evaluate viral dispersions in the extremely low-concentration range (e.g., ~10^3^ copies/mL), where efficient virus capture and enrichment are required. However, quantitative evaluation in this range becomes difficult due to large measurement errors near the detection limit. Therefore, we determined the parameters of the kinetic model based on experiments using viral dispersions in a low-concentration range (10^5^ copies/mL) where reliable quantification is attainable, and adopted an approach that extrapolates the results to the extremely low-concentration regime, in which the encounter probability between the target and magnetic particles becomes the dominant factor. This study aims to define the operational limits for rapid virus capture, providing critical insights for the development of next-generation antigen tests suitable for POC applications in future pandemics.

## 2. Materials and Methods

Virus preparation and cell culture were performed as described below. Madin–Darby canine kidney (MDCK) cells (accession no. CCL-34, ATCC, Manassas, VA, USA) were cultured in Dulbecco’s Modified Eagle’s Medium (Fujifilm Wako Pure Chemical Corporation, Osaka, Japan) supplemented with 10% fetal bovine serum (Fujifilm Wako Pure Chemical Corporation), 100 µg/mL streptomycin (Fujifilm Wako Pure Chemical Corporation), and 100 U/mL penicillin. The conditions in a CO_2_ incubator were maintained at 37 °C in a 5% CO_2_ atmosphere. IAVs (Puerto Rico/8/1934/H1N1) were propagated in MDCK cells and purified by super centrifugation at 200,000× *g*. The aliquots were stored as IAV stocks (~10^8^ TCID_50_/mL, ~4 × 10^7^ copies/mL) at −80 °C. The titer and copy number of the IAV stocks were determined using the 50% tissue culture infectious dose (TCID_50_) assay [31,32] and real-time reverse transcription qPCR (RT-qPCR) assay [33,34], respectively.

A magnetic particle (Sera-Mag^TM^ SpeedBead Carboxylate-Modified Magnetic Particles, ~1000 nm in diameter, Global Life Sciences Technologies Japan, Tokyo, Japan) was modified with an anti-IAV H1N1 hemagglutinin (HA) monoclonal antibody (11684-R016, Sino Biological Inc., Beijing, China). The modification of the magnetic particles with the anti-HA antibodies was performed by amine coupling [35,36]. An amine coupling kit containing 1-ethyl-3-(3-dimethylaminopropyl)carbodiimide (EDC) and *N*-hydroxysuccinimide (NHS) was purchased from Dojindo Laboratories (Kumamoto, Japan). For bead preparation, 50 μL EDC and NHS solutions at a concentration of 100 mM were mixed, and the mixture was immediately added to 100 μL of magnetic beads 25 mg/mL in MES buffer (pH 6.0). The mixture was incubated for 30 min at room temperature to activate the carboxyl group. The beads were washed with 25 mM acetic acid three times using magnetic separation and redispersed in 50 μL of acetic acid, followed by the addition of 50 μL of the anti-hemagglutinin antibody 0.5 mg/mL in PBS. The antibodies were immobilized on the surface of the beads by incubating the mixture for 2 h at 20 °C with shaking (1500 rpm) on a block bath shaker (MyBL-100CS; AS ONE, Osaka, Japan). After the modification process, an oligo amine-terminated PEG reagent (Blockmaster CE210, JSR Life Sciences Co., Tokyo, Japan) and Tris solution were used to block the functional groups on the surfaces of the AM-MPs by sequential incubation—first with the PEG reagent overnight at 4 °C, followed by incubation with Tris for 1 h at 20 °C—under shaking.

The AM-MPs were purified by magnetic separation and dispersed in 10 mM HEPES buffer (HB, 1 M HEPES-NaOH pH 7.9, Tamagawa Seiki Co., Ltd., Nagano, Japan, diluted 100-fold with Milli-Q water before use).

The RT-qPCR assay was conducted to measure the IAV copy number. Viral RNA was isolated from a 200 µL solution using an automated nucleic acid extraction system (magLEAD 12gC, Precision System Science Co., Ltd., Chiba, Japan) according to the manufacturer’s instructions. Each RNA was eluted in 50 µL of an elution buffer. The isolated RNA was immediately subjected to RT-qPCR with the TaqMan Fast Virus 1-Step Master Mix (Thermo Fisher Scientific K.K., Tokyo, Japan), a TaqMan probe (5′-FAM-ATYTCGGCTTTGAGGGGGCCTG-MGB-3′), and a primer pair (Forward: 5′-CCMAGGTCGAAACGTAYGTTCTCTCTATC-3′, Reverse: 5′-TGACAGRATYGGTCTTGTCTTTAGCCAYTCCA-3′). The probe and primer sequences were derived from the protocols of IAV detection released by the World Health Organization (WHO) [31]. Thermal cycling was carried out on the LightCycler 96 (Roche Diagnostics K.K., Tokyo, Japan) for reverse transcription at 50 °C for 5 min, denaturation of the reverse transcription polymerase at 95 °C for 20 s, and 40 cycles of PCR at 95 °C for 3 s and 60 °C for 30 s. Copy number quantification was carried out with an IAV standard curve. An assay was performed in duplicate.

**Figure 1:**
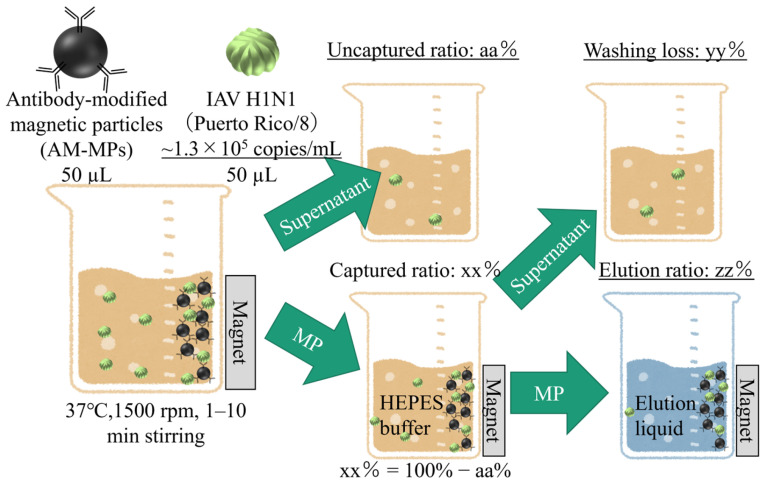
Schematic of the sample preparation protocol to estimate the ratios of viral capturing, washing loss, and elution. The AM-MPs were mixed with the thawed IAV stocks and stirred at 37 °C and 1500 rpm by a block bath shaker. Three times magnetic separations and twice dispersion processes were performed. Each supernatant of the magnetic separations was used for the viral RNA isolation and RT-qPCR. The concentrations of the AM-MPs and virus solutions indicated in the figure were typical numbers of them in this study.

The sample solutions for the viral RNA isolation were prepared by the protocol below. Figure 1 shows the schematic of the protocol. The thawed IAV stocks were 300-fold diluted by phosphate-buffered saline (PBS, 10× PBS (-) 163-25265, Fujifilm Wako Pure Chemical Corporation, diluted 10-fold with Milli-Q water before use). Some portions of the diluted IAV stocks (50 µL) were mixed with the AM-MPs dispersions (50 µL). The 100 µL of mixtures were stirred at 37 °C and 1500 rpm by a block bath shaker (MyBL-100CS, ASONE, Osaka, Japan) to capture viruses by the AM-MPs. The AM-MPs with captured viruses were separated from supernatants by magnetic separation using a magnet stand. The supernatants were used for the viral RNA isolation and RT-qPCR assay to estimate the efficiency of virus capturing. The AM-MPs with captured viruses were washed with 100 µL HB and were separated from the supernatants by magnetic separation. The washing supernatants were used for the viral RNA isolation and RT-qPCR assay to estimate the ratio of washing loss. The washed AM-MPs were dispersed with elution liquids including a 5 M guanidinium thiocyanate aqueous solution. The AM-MPs dispersed in the elution liquids were separated from the supernatants by magnetic separation. The supernatants of the elution liquids were used for the viral RNA isolation and RT-qPCR assay to estimate the efficiency of the elution. Each 100 µL supernatant was mixed with 100 µL of the elution liquid, and each 200 µL mixture was set into the magLEAD 12gC.

## 3. Results

### 3.1. Estimation of the Viral Collection Efficiency

**Figure 2:**
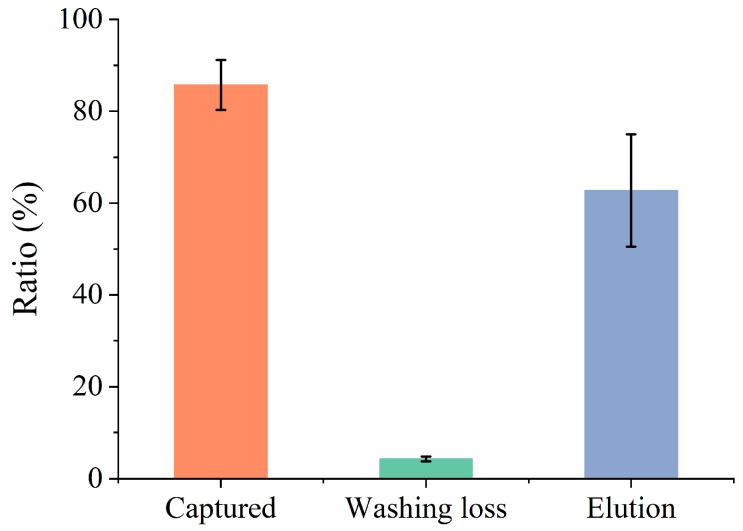
Viral collection efficiency at each step of the protocol. Each ratio was calculated using the copy numbers of the supernatants obtained during magnetic separation and the diluted IAV stock. Error bars represent standard deviations (*n* = 3).

The viral collection efficiency at each step of the protocol was assessed based on measurements of the captured ratio (calculated as 1 − uncaptured ratio), washing loss, and elution ratio. The corresponding results are presented in Figure 2. The final concentrations of AM-MPs and the diluted IAV stock in the mixtures were 2.5 × 10^8^ particles/mL and 6.5 × 10^4^ copies/mL, respectively. The mixture was stirred for 10 min during the capturing step. Hereafter, this stirring duration in the capturing step is defined as “reaction time”.

Approximately 86% of the viruses in the diluted IAV stock were captured by the AM-MPs. In contrast, washing loss remained consistently low, at approximately 4% of the diluted IAV stock. However, the elution ratio was unstable and markedly smaller than the difference between the captured ratio and washing loss. Preliminary results indicated that viral loss during the elution process increased markedly when BSA was used to mask the AM-MPs. This loss may have resulted from non-specific adsorption to the AM-MP surface, influenced by guanidinium thiocyanate. Therefore, in this study, we focused on the captured ratio to evaluate viral collection efficiency.

### 3.2. Influence of AM-MP Concentration on Viral Collection Efficiency

**Figure 3:**
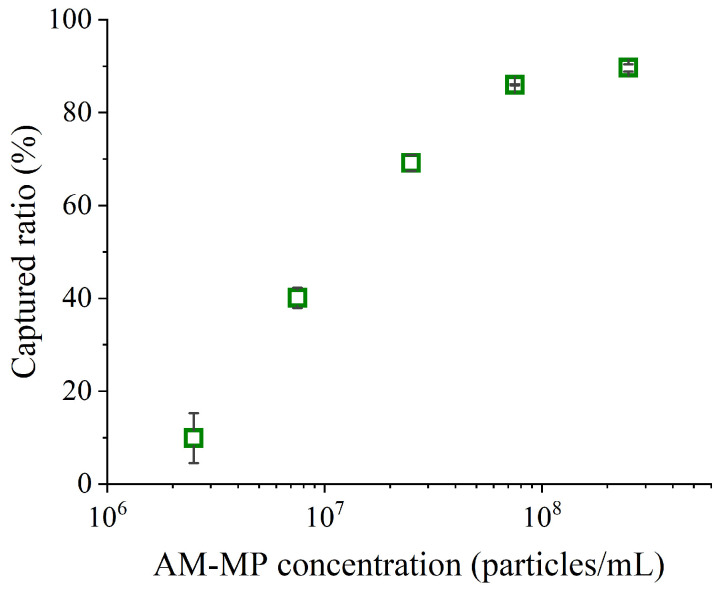
Captured ratio at each AM-MP concentration. The reaction time was 10 min. Each captured ratio was calculated using the copy numbers of the supernatants obtained during magnetic separation and the diluted IAV stock. Error bars represent standard deviations (*n* = 2).

The captured ratios at various AM-MP concentrations were measured to assess differences in viral collection efficiency. The results are presented in Figure 3. AM-MP concentrations in the mixtures were 2.5 × 10^6^, 7.5 × 10^6^, 2.5 × 10^7^, 7.5 × 10^7^, and 2.5 × 10^8^ particles/mL, while the final concentration of the diluted IAV stock was 6.5 × 10^4^ copies/mL. The reaction time was 10 min.

When AM-MP concentrations exceeded 10^8^ particles/mL, more than 80% of IAVs were captured by the AM-MPs. In contrast, at concentrations below 10^8^ particles/mL, the AM-MPs captured only a small fraction of IAVs within 10 min. Although the lowest AM-MP concentration (2.5 × 10^6^ particles/mL) was close to the typical bead concentration used in digital ELISA [20], it was insufficient for high-efficiency capture within 10 min.

### 3.3. Influence of Reaction Time on Viral Collection Efficiency

**Figure 4:**
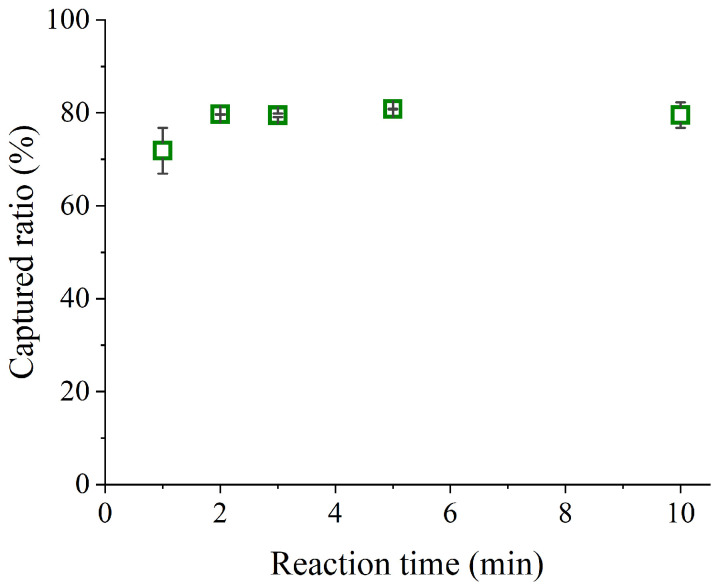
Captured ratio at each reaction time. The AM-MPs were diluted in HB to 2.5 × 10^8^ particles/mL. Each captured ratio was calculated using the copy numbers of the supernatants obtained during magnetic separation and the diluted IAV stock. Error bars represent standard deviations (*n* = 2).

The captured ratios at different reaction times (1–10 min) were measured to evaluate variations in viral collection efficiency. The results are shown in Figure 4. The final AM-MP concentration was 2.5 × 10^8^ particles/mL, and the final concentration of the diluted IAV stock was 6.5 × 10^4^ copies/mL. The mixture was stirred for the specified duration during the capturing step.

When reaction times exceeded 2 min, approximately 80% of IAVs were captured by the AM-MPs. In contrast, at 1 min binding, the AM-MPs captured approximately 70% of IAVs. This result indicates that the captured ratio of IAV reaches saturation within 2 min when the AM-MP concentration is 2.5 × 10^8^ particles/mL. However, considering the experimental conditions shown in Figure 2 and Figure 3, where the captured ratio of IAV could reach approximately 90%, saturation at around 80% appears inconsistent. Preliminary investigations revealed that the elapsed time between thawing the original IAV solution and mixing with AM-MPs strongly influences the upper limit of the captured ratio. When the solution was prepared and mixed with AM-MPs immediately after thawing, the captured ratio reached approximately 90%. In contrast, when mixing occurred 2 h after thawing, the upper limit decreased to about 80%, and after 24 h, it further declined to approximately 60%. This phenomenon is likely attributable to structural damage to the viral envelope during thawing, followed by progressive disintegration and lysis over time, which increases the amount of nucleic acids that cannot be recovered by AM-MPs.

## 4. Discussion

Based on the experimental results obtained, we performed an analysis grounded in reaction kinetics. Following the approach in prior work [36], we modeled the binding and dissociation between AM-MPs and virus particles (e.g., IAV virions) mediated by antigen–antibody interactions. Under the experimental conditions of this study, we assume that the initial concentration of AM-MPs, [*MP*]_0_, is sufficiently higher than that of virus particles, [*Virion*]_0_, i.e., [*Virion*]_0_ << [*MP*]_0_. Since both AM-MPs and virus particles possess multiple binding sites, complexes could theoretically form in which several AM-MPs bind to a single virus particle or multiple virus particles bind to a single AM-MP. For simplicity, however, this study considers only complexes composed of one AM-MP and one virus particle. Under the condition [*Virion*]_0_ << [*MP*]_0_, the vast majority of AM-MPs remain unbound, and at most one virus particle associates with a single AM-MP. Although multiple AM-MPs may bind to a single virus particle, the impact on the overall AM-MP concentration is negligible under these conditions. Therefore, restricting the analysis to one-to-one binding between AM-MPs and virus particles is considered sufficient. Then, the concentration of complexes formed between AM-MPs and virus particles at time *t* after the initiation of the binding reaction, [*MP* − *Virion*], can be described by the following differential equation:

(1) $$\frac{d\left[MP-Virion\right]}{dt}=k_{a}\left[MP\right]\left[Virion\right]-k_{d}\left[MP-Virion\right]$$

where *k*_a_ and *k*_d_ denote the association and dissociation rate constants, respectively. Under the condition [*Virion*]_0_ << [*MP*]_0_, solving Equation (1) yields the capture efficiency *P* at the reaction time *t*, defined as *P* = [*MP* − *Virion*]/[*Virion*]_0_:

(2) $$P=\frac{{k_{a}\left[MP\right]}_{0}}{{k_{a}\left[MP\right]}_{0}+k_{d}}\left(1-exp\left(-\left({k_{a}\left[MP\right]}_{0}+k_{d}\right)t\right)\right)$$

As shown in Equation (2), the capture efficiency *P* depends on three parameters: [*MP*]_0_, *k*_a_ and *k*_d_. Since [*MP*]_0_ is given by the experimental conditions, determining *k*_a_ and *k*_d_ for the reaction system enables calculation of *P* at any given reaction time *t*.

**Figure 5:**
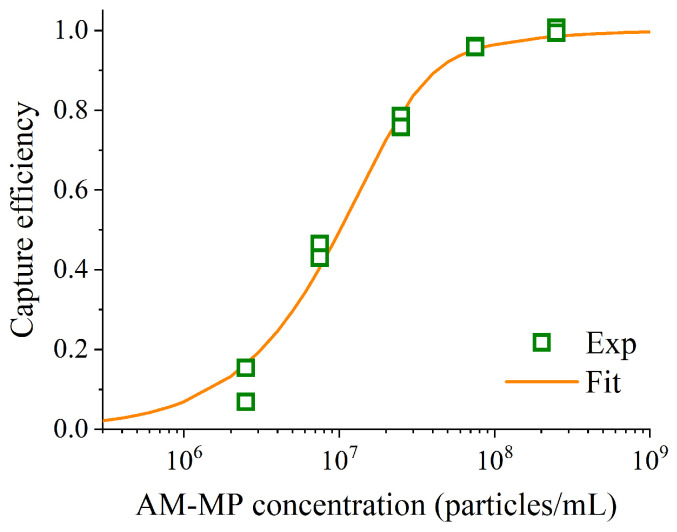
Experimental data (Exp) and a regression curve (Fit) for the ratio of capture efficiency to saturation capture efficiency (*P*/*P*ₛₐₜ) at each AM-MP concentration. The reaction time was 10 min.

Least-squares fitting of Equation (2) was applied to the experimental data shown in Figure 3, obtained by varying the concentration of AM-MPs, using OriginPro 2018b (OriginLab Corporation, Northampton, MA, USA). This fitting enabled estimation of *k*_a_ and *k*_d_ for the reaction system. As noted in Section 3.3, the experimentally evaluated captured ratio did not reach 100% but instead saturated at a certain value (e.g., approximately 80%) in each experiment. This is likely attributable to the presence of nucleic acids derived from disrupted virus particles in the sample, which cannot be captured by AM-MPs. To account for this, we normalized the captured ratio by its saturation value and used the normalized values for theoretical analysis. The fitting results for the experimental data in Figure 3 are shown in Figure 5. As shown in Figure 4, saturation was achieved at a reaction time of 10 min with AM-MP concentration of 2.5 × 10^8^ particles/mL. Therefore, the saturation captured ratio used in the analysis of Figure 5 was set to the captured ratio at the concentration of 2.5 × 10^8^ particles/mL. The ratio of the captured ratio to the saturation value was fitted using Equation (2). The fitting resulted in *k*_a_ = 8.3 × 10^4^ M^−1^ s^−1^ and *k*_d_ = 5.1 × 10^−4^ s^−1^, with a coefficient of determination of R^2^ = 0.9870. The obtained *k*_a_ value was approximately four times higher than the theoretical estimate assuming no stirring, calculated using the equation for *k*_a_ estimation described in prior work [36]. Given that the binding reaction in this study was conducted under stirring, this elevated *k*_a_ likely reflects the rate enhancing effect of stirring. In addition, the *k*_d_ value falls within the typical range reported for monoclonal antibodies (10^−2^–10^−4^ s^−1^) [37], indicating that it is a reasonable and consistent estimate.

**Figure 6:**
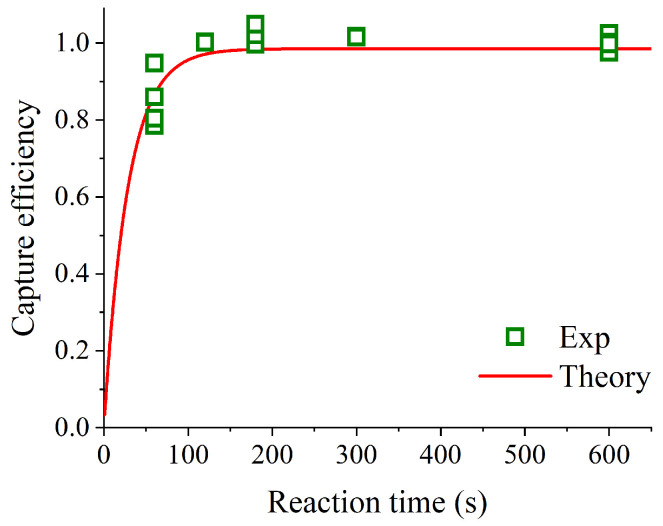
Experimental data (Exp) and theoretical curve (Theory) for the ratio of capture efficiency to saturation capture efficiency (*P*/*P*ₛₐₜ) at each reaction time. The concentration of AM-MPs was 2.5 × 10^8^ particles/mL.

Next, the experimental data obtained by varying the reaction time were compared with the theoretical curve calculated from Equation (2) using the *k*_a_ and *k*_d_ values obtained from the fit in Figure 5. The comparison between the experimental data and the theoretical curve is presented in Figure 6. For this comparison, we supplemented the data shown in Figure 4 with additional measurements at reaction times of 1, 3, and 10 min (*n* = 2). The saturation captured ratio used in Figure 6 was set to the captured ratio at the reaction time of 10 min. The coefficient of determination for the comparison was R^2^ = 0.9981, demonstrating excellent agreement between theory and experiment. This result corroborates the observation described in Section 3.3 and shown in Figure 4—namely, that at an AM-MP concentration of 2.5 × 10^8^ particles/mL, the saturation captured ratio is reached within only 2 min. Under high AM-MP concentrations such as those used in this study (e.g., ≥10^8^ particles/mL), rapid capture of virus particles is feasible, suggesting that AM-MP–based immunoassays can be substantially accelerated. Furthermore, because theoretical predictions based on Equation (2) closely reproduce the experimental data, Equation (2) can be used to estimate the minimum reaction time sufficient for effective virus capture at each AM-MP concentration, thereby enabling efficient optimization of capture reaction conditions. However, the influence of viral particle concentration in the experiments requires careful consideration. In the present study, to quantify by qPCR, the IAV sample concentration mixed with AM-MPs was set not in the extremely low-concentration range near the detection limit (e.g., ~10^3^ copies/mL)—where measurements would become unreliable—but in a low-concentration range (10^5^ copies/mL) that still leaves a sufficient amount of virus for qPCR quantification even when the recovery efficiency of AM-MPs is high. In both concentration ranges, the condition assumed in the theoretical model, [*Virion*]_0_ << [*MP*]_0_, is sufficiently satisfied; therefore, Equation (2) is considered applicable. As indicated in Figure 5, the capture efficiency is expected to be governed by the encounter probability, which is predominantly determined by [*MP*]_0_. In the extremely low-concentration regime as well, Equation (2) predicts that—similar to the experimentally tested low-concentration regime—the encounter probability becomes insufficient unless [*MP*]_0_ is maintained at a sufficiently high level (≥10^8^ particles/mL), leading to inadequate capture efficiency. The kinetic model developed in this study is therefore useful for estimating the AM-MP concentration required to achieve such conditions and for predicting the practical limits for shortening the reaction time.

Based on the relationship between particle concentration and viral capture efficiency elucidated in this study, we compared previously reported data on magnetic particle concentration and reaction time for d-ELISA [20,38], CLEIA [39,40], and the multiparticle-concentrated digital immunoassay (MCDIA), a kind of digital immunoassay developed by the authors [36,41]. A summary of this comparison is provided in Table 1. In d-ELISA, the number of AM-MPs per well is limited to at most one, thereby restricting the available AM-MP concentration to approximately 10^6^ particles/mL, as determined by the total number of microwells in the array. As a result, the reaction time typically ranges from 30 to 60 min, making rapid detection difficult. In contrast, CLEIA accommodates a broader range of AM-MP concentrations, with reported values spanning 10^5^ to 10^8^ particles/mL. Notably, when the AM-MP concentration reaches the order of 10^8^ particles/mL, the reaction time can be reduced to as little as 5 min. These trends are consistent with the findings of the present study. Our MCDIA system achieves a reaction time of 10 min using AM-MP concentrations on the order of 10^8^ particles/mL, and the results presented in this study corroborate that the reaction time could be further reduced to approximately 2 min. Overall, the insights obtained in this study offer important guidelines for the design and protocol optimization of next-generation rapid and highly sensitive immunoassay systems.

## 5. Conclusions

This study demonstrated that high concentrations (≥10^8^ particles/mL) of antibody-modified magnetic particles (AM-MPs) enable rapid and efficient capture of influenza A virions, achieving saturations of the captured ratio within 2 min. These experimental findings were well supported by kinetic modeling, which well described the observed trends and provided a rational basis for optimizing assay conditions. Furthermore, our reaction-kinetics-based inference indicated that, unless AM-MPs are used at high concentrations on the order of ≥10^8^ particles/mL, the effective encounter rate between AM-MPs and low or extremely low concentrations of targets (e.g., 10^3^–10^5^ copies/mL) becomes insufficient, thereby preventing rapid target capture within short assay times. As a limitation of the applicability of the present model, it should be noted that our experimental validation was performed only in the low-concentration range of 10^5^ copies/mL. Nevertheless, in the extremely low-concentration regime, the condition assumed in the theoretical model, [*Virion*]_0_ << [*MP*]_0_, is similarly (or even more strictly) satisfied. Accordingly, these results will provide practical guidelines for the rapid development of sensitive bead-based immunoassays using another antibody.

## Figures and Tables

**Table 1:** Relationship between particle concentration and reaction time in previous reports.

Method	AM-MP Concentration	Reaction Time
Digital ELISA [20]	1.44 × 10^6^ particles/mL	60 min
Digital ELISA (Simoa) [38]	4 × 10^6^ particles/mL	30 min
CLEIA [39]	~3 × 10^5^ particles/mL	30 min
CLEIA [40]	~2 × 10^8^ particles/mL	5 min
MCDIA [36,41]	2.5 × 10^8^ particles/mL	10 min
Present work ^1^	2.5 × 10^8^ particles/mL	2 min

^1^ No detection system was constructed in this study.

## Data Availability

The datasets presented in this article are not readily available because the data are part of an ongoing study, and due to technical/time limitations.
